# Fine-tuning the expression of target genes using a *DDI2* promoter gene switch in budding yeast

**DOI:** 10.1038/s41598-019-49000-8

**Published:** 2019-08-29

**Authors:** Yong Wang, Kaining Zhang, Hanfei Li, Xin Xu, Huijun Xue, Pingping Wang, Yu V. Fu

**Affiliations:** 1 State Key Laboratory of Microbial Resources, Institute of Microbiology, Chinese Academy of Sciences, Beijing, 100101 China; 2 Savaid Medical School, University of Chinese Academy of Sciences, Beijing, 100101 China; 3 Qingdao Baihuizhiye Biotech Co.Ltd, Qingdao, 266109 China

**Keywords:** Gene regulation, Applied microbiology

## Abstract

Tuned gene expression is crucial to the proper growth and response to the environmental changes of an organism. To enable tunable gene expression as designed is desirable in both scientific research and industrial application. Here, we introduce a novel promoter switching method based on the *DDI2* promoter (P_*DDI2*_) that can fine tune the expression of target genes. We constructed a recyclable cassette (P_*DDI2*_-*URA3*-P_*DDI2*_) and integrated it upstream of yeast target genes to replace the native promoters by *DDI2* promoter without introducing any junk sequence. We found that the presence or absence of cyanamide as an inducer could turn on or off the expression of target genes. In addition, we showed that P_*DDI2*_ could act as a gene switch to linearly regulate the expression levels of target genes *in vivo*. We switched the original promoters of *RAD18*, *TUP1*, and *CDC*6 with P_*DDI2*_ as a proof-of-concept.

## Introduction

For each cell, the transcriptional programs are modified to maintain specific intracellular conditions to ensure optimal growth and function^1,2^. When environmental conditions change suddenly, the ability of cells to rapidly adjust genome expression is critical for competitive fitness and cell survival^3,4^. Unicellular organisms like *Saccharomyces cerevisiae* have evolved autonomous mechanisms in response to changes in the environment during organismal development^2,5^. Among them, the promoter is the most basic and the most important tool to control gene expression programs. Various strengths of constitutive and inducible promoters provide a broad range of genetic control in *S. cerevisiae*^6,7^.

Constitutive promoters maintain relatively stable expression levels and do not require inducers or repressors^8,9^. The widely used constitutive promoters in budding yeast regulate genes involved in the glycolytic pathway, such as the promoters of alcohol dehydrogenase 1 (P_*ADH1*_)^10^ and phosphoglycerate kinase (P_*PGK*_)^11^. Inducible promoters are more suitable than constitutive promoters for regulating gene expression in response to stimuli, which may be desirable for the fine-tuning purpose. The use of inducible promoters is limited by the strength of the promoter’s response to the inducer, the leak degree of the expression controlled by the promoter, and the cost of induction^9^. In yeast, the most commonly used tightly-inducible promoters are P_*GAL1*_^12^, P_*GAL10*_, and P_*GAL7*_^13,14^, which are induced by galactose and strongly repressed in glucose medium^15^. Yeast cells prefer glucose and fructose as carbon sources, thus when P_*GAL1*_ is used to induce target gene expression, the original carbon source must be replaced by galactose. The process of changing medium is difficult and galactose is considered too expensive for use in large-scale cultures^8^. P_*CUP1*_ is another commonly used inducible promoter in budding yeast, which is activated by Cu^2+,^^16^. Compared to P_*GAL1*_ promoter, the P_*CUP1*_ promoter displays rather high basal level expression in the absence of Cu^2+ ^^17,18^. Moreover, Cooper could be enriched inside the cells that make a serious impact on both cellular structures and metabolisms. The tetracycline regulatory system (Tet-on and Tet-off), which is originally from bacteria, has been widely used to regulate gene expression in eukaryotes^19^. In eukaryotic cells, this system has been applied to control the RNA polymerase III-driven transcription of eukaryotic tRNA genes^20,21^. However, this system requires the introduction of heterogeneous regulatory proteins into host cells^19,22^. Therefore, simple and efficient inducible promoters are needed in *S. cerevisiae*.

The *DDI2* and *DDI3* genes were reported to display the highest induction (>100-fold) in yeast cells after treatment with the DNA-damaging agent methyl methanesulfonate (MMS)^23^. *DDI2* and *DDI3* are two identical genes with exactly the same ORF sequences and only one nucleotide difference in their promoters^24^, but they are located on different chromosomes. Protein sequence analysis and experimental verification revealed that *DDI2/3* encode a cyanamide hydratase in *S. cerevisiae*^25–27^. Meanwhile, it has been reported that cyanamide can highly induce the expression of *DDI2/3* as MMS^24,28^. Cyanamide is a clean fertilizer because in the air it can be converted naturally into ammonia and carbon dioxide^24^. Cyanamide is also used as an alcohol deterrent drug^29,30^ and as a raw compound in the pharmaceutical industry to produce guanidine derivatives^31^. Hence, we propose to utilize the *DDI2* promoter to develop a novel gene expression regulating system induced by cyanamide.

Here, we used the superfolder green fluorescent protein gene (*sfGFP*) as a reporter gene to determine the strength and regulation of P_*DDI2*_ compared with three other widely-used promoters (P_*ADH1*_, P_*CUP1*_ and P_*GAL1*_)^32^. Quantitative analysis results demonstrated that the induction level of P_*DDI2*_ is strong and leaky level is low. As a proof-of-concept, we replaced the native promoters of three yeast genes (*RAD18*, *TUP1*, and *CDC*6) with P_*DDI2*_, without redundant DNA sequence left. As anticipated, P_*DDI2*_ showed tunable control of the target genes, especially the regulation of the essential gene *CDC*6 as a yeast safeguard switch. We demonstrated a linear gene expression control on the target gene correlated with the inducing strength, which further facilitates the precise regulation of gene expression in budding yeast.

## Results

### Optimal *DDI2* promoter induction time and inducer concentration

**Figure 1:**
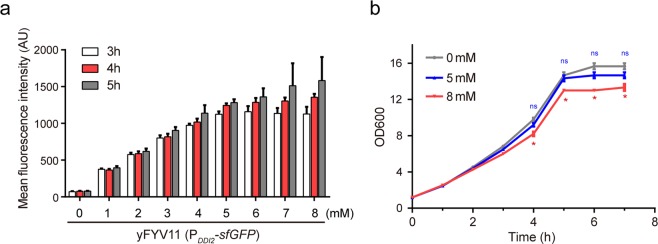
Optimal *DDI2* promoter induction time and concentration analysis. (**a**) Mean fluorescence intensity of the yFYV11 (P_*DDI2*_*-sfGFP*) strain under the control of P_*DDI2*_ at different induction time points (3–5 h) and different cyanamide concentrations (0–8 mM) observed by flow cytometry. (**b**) Effect of cyanamide on the growth rate of yeast cells. After adding different concentrations of cyanamide (0, 5, and 8 mM), OD600 values were measured every hour for 7 h. The 5 mM cyanamide hardly affected the growth rate of the yeast cells (ns: not significant), however, 8 mM cyanamide affected BY4741 cell growth (**p < 0.01). (t test, n = 3, *p < 0.05, **p < 0.01, ***p < 0.001).

P_*DDI2*_ is a novel inducible promoter that is efficiently induced by cyanamide^24,28^. We tested whether the strength of P_*DDI2*_ is affected by both the induction time and inducer concentration. To determine the optimal induction time and cyanamide concentration, we established a reporter gene system in which an *sfGFP* gene is under the control of *DDI2* promoter (see Supplementary Fig. S1a). To avoid the potential effect from gene copy number variations a single-copy plasmid YCp (yeast centromere plasmid) was chosen as the backbone vector^33^. We measured the expression of the P_*DDI2*_*-sfGFP* reporter gene at different induction time points (3–5 h) and cyanamide concentrations (0–8 mM) by flow cytometry. As shown in Fig. 1a, the mean fluorescence values of *sfGFP* increase with increasing cyanamide concentrations (0–8 mM) and stabilize when cyanamide concentrations are 5 mM and above. The highest *sfGFP* expression level is observed after 5-hour induction among 3–5 hour induction time points, which implies that P_*DDI2*_ activity is proportional to the induction time.

It has been reported that cyanamide has modest toxicity to humans^34^. To investigate the effect of cyanamide toxicity on the growth of yeast cells, we generated growth curves for wild-type *S. cerevisiae* cells grown in YPD medium supplemented with 0, 5 and 8 mM cyanamide. Figure 1b shows that the growth rate of yeast cells is hardly affected by 5 mM cyanamide, but with 8 mM cyanamide the cell concentration (OD600) is slightly affected and at longer induction time the cell growth rate is lower than at 0 and 5 mM cyanamide concentrations. These data suggest that P_*DDI2*_ is induced efficiently by cyanamide and has high promoter activity and that cyanamide has little effect on cell growth at concentrations equal to or lower than 5 mM.

### Comparison of the *DDI2* promoter with *ADH1*, *CUP1* and *GAL1* promoters

**Figure 2:**
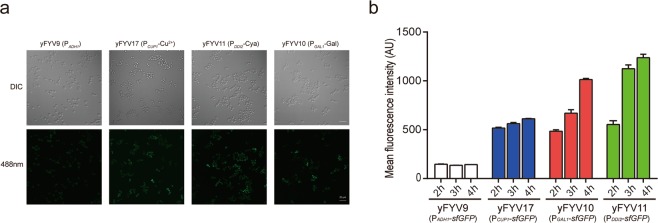
*sfGFP* expression under the control of four promoters. (**a**) Comparison of yFYV9 (P_*ADH1*_*-sfGFP*), yFYV17 (P_*CUP1*_*-sfGFP*), yFYV10 (P_*GAL1*_-*sfGFP*), and yFYV11 (P_*DDI2*_-*sfGFP*) fluorescence intensities under the control of P_*ADH1*_, P_*CUP1*_, P_*GAL1*,_ or P_*DDI2*_. The yFYV11 (P_*DDI2*_-*sfGFP*) strain was induced by 5 mM cyanamide (Cya) for 3 h, the yFYV10 (P_*GAL1*_-*sfGFP*) strain was induced in galactose (Gal) medium for 3 h and the yFYV17 (P_*CUP1*_*-sfGFP*) strain was induced in 0.5 mM Cu^2+^ medium for 3 h. (**b**) Mean fluorescence changes of yFYV9 (P_*ADH1*_-*sfGFP*), yFYV17 (P_*CUP1*_-*sfGFP*) and yFYV10 (P_*GAL1*_-*sfGFP*) strains at different time points by flow cytometry. The yFYV10 (P_*GAL1*_*-sfGFP*) strain was induced in galactose (Gal) medium for 2–4 h and the yFYV17 (P_*CUP1*_*-sfGFP*) strain was induced in the medium containing 0.5 mM CuSO_4_ for 2–4 h.

We compared the strength and induction process of P_*DDI2*_ with three classic promoters, P_*ADH1*_, P_*CUP1*_ and P_*GAL1*_. To facilitate the comparisons, we established the same reporter gene system as described above just by replacing *DDI2* promoter with three other promoters. The four plasmids carrying P_*DDI2*_*-sfGFP*, P_*ADH1*_*-sfGFP*, P_*CUP1*_*-sfGFP* and P_*GAL1*_*-sfGFP* (see Supplementary Fig. S1a–d) were transferred to the *S. cerevisiae* cells. After the yeast cells were induced by corresponding inducer or water for 3 hours, we irradiated cells with the 488 nm laser to observe sfGFP signal by fluorescence microscopy. As shown in Fig. 2a, the fluorescence intensity of yFYV11 (P_*DDI2-sfGFP*_) cells is much greater than that of yFYV9 (P_*ADH1-sfGFP*_) cells. Comparing the three inducible promoters, the yFYV11 (P_*DDI2-sfGFP*_) cells show higher fluorescence intensity than both yFYV10 (P_*GAL1-sfGFP*_) and yFYV17 (P_*CUP1-sfGFP*_) cells, (Fig. 2a).

To get quantitive data to confirm these observations, we measured the expression of *sfGFP* using flow cytometry in different conditions. For yFYV17 (P_*CUP1*_*-sfGFP*) cells, a final concentration of 0.5 mM CuSO_4_ was applied for induction according to previous study^35^, then the mean fluorescence values were measured at different time points. yFYV10 (P_*GAL1*_*-sfGFP*) cells were grown to log phase in glucose medium, then washed and incubated in raffinose medium for 3 hours, which does not repress or induce transcription of the *GAL1* promoter^36^, prior to inducing in the galactose medium for 2–4 hours as described in the previous reports^37–39^. The yFYV11 (P_*DDI2*_*-sfGFP*) cells were directly induced by 5 mM cyanamide for 2–4 hours.

As it is expected, the mean fluorescence values for yFYV9 (P_*ADH1*_*-sfGFP*) cells are relatively low and stable at different time points (Fig. 2b). At the 4-hour time point, compared the mean fluorescence values of yFYV11 (P_*DDI2*_*-sfGFP*) cells induced with 5 mM cyanamide to that of yFYV9 (P_*ADH1*_*-sfGFP*) cells and yFYV17 (P_*CUP1*_*-sfGFP*) induced by 0.5 mM CuSO_4_, P_*DDI2*_ exhibited upwards of an 8.72-fold and a 2.03-fold increase in mean fluorescence over P_*ADH1*_ and P_*CUP1*_. After 4-hour induction, the mean fluorescence of yFYV10 (P_*GAL1*_*-sfGFP*) reaches a rather high level. However, the mean fluorescence of P_*DDI2*_ is still 1.23-fold higher than that of P_*GAL1*_ at this time point (Fig. 2b). These results suggest that P_*DDI2*_ has significantly higher activity than P_*ADH1*_, P_*CUP1*_, and P_*GAL1*_ at the same induction time. Hence, P_*DDI2*_ can be used as a novel promoter with high inducibility.

### Switching the native promoters of target genes *RAD18* and *TUP1* with *DDI2* promoter

P_*DDI2*_ can be induced by cyanamide, therefore, it is reasonable that the native promoter of a target gene on its genomic locus could be replaced with P_*DDI2*_ to regulate its expression. As a proof-of-concept, we first replaced the promoters of two target genes (*RAD18* and *TUP1*) with P_*DDI2*_. *RAD18* plays an important role in DNA damage repair caused by UV or methyl methanesulfonate^23,40^. *TUP1* is a general repressor of transcription in yeast, and a *tup1Δ* strain exhibits an obvious clumpy cell morphology phenotype^41,42^ that can be observed directly.

We used the recyclable cassettes method reported previously^37,43^ to replace the native promoter in the yeast genome by one-step transformation followed by two-step selection. First, the P_*DDI2*_-*URA3*-P_*DDI2*_ (DUD) cassettes were amplified by PCR to replace the promoter of the target gene by homologous recombination. Secondly, the cassette-integrated strain was obtained using auxotrophic plates (SD-*URA*). The most important step is that one copy of the P_*DDI2*_ and *URA3* deleted cells are selected on 5-FOA plates so that only one P_*DDI2*_ regulating the target gene is obtained without redundant DNA. For more efficient promoter shuffling, the several hundred base pairs flanking the homology arms were generated by overlapping PCR.

**Figure 3:**
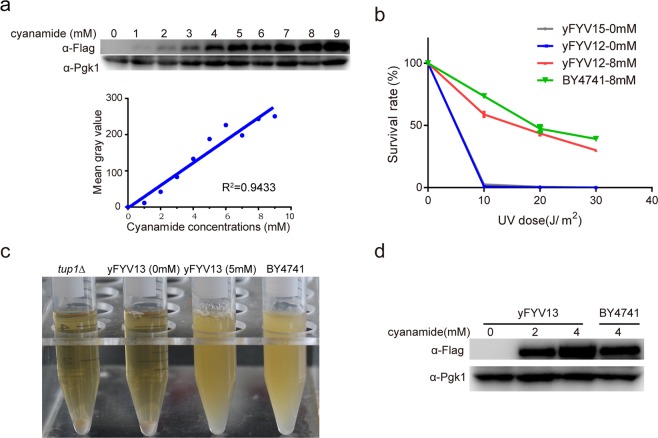
Switching the target gene promoters with the *DDI2* promoter *in vivo*. (**a**) yFYV12 (P_*DDI2*_-*RAD18-*5 × *FLAG*) cells were induced with 0–9 mM cyanamide for 4 h, then the total protein was extracted. The abundance of the Rad18 protein was measured by western blot analysis using an anti-Flag antibody. The Pgk1 protein was used as an internal loading control. The mean gray value of Rad18 in the western blot was quantified using Image J software (1.50i). The image of blots is cut from the image of Full-length blots, which are presented in Supplementary Fig. S2. The linear equation (y = 30.16x − 2.67) fitted to the change of gray values. (**b**) Survival rates after UV treatment of strains BY4741, yFYV15 (*rad18Δ*), and yFYV12 (P_*DDI2*_-*RAD18*) with or without cyanamide. (**c**) yFYV16 (*tup1Δ*), BY4741, and yFYV13 (P_*DDI2*_-*TUP1*) cells were incubated overnight with 0 or 5 mM cyanamide, and then the cultures were imaged. (**d**) yFYV13 (P_*DDI2*_-*TUP1*) cells were induced by cyanamide (0, 2, or 4 mM) for 4 h, then the total protein was extracted from yFYV13 and BY4741. Tup1 protein abundance was measured by western blot analysis using an anti-Flag antibody. The image of blots is cut from the image of Full-length blots, which are presented in Supplementary Fig. S3.

To confirm that P_*DDI2*_ could switch on/off gene expression on the yeast genome, we labeled the two target genes (*RAD18* and *TUP1*) with 5 × Flag tag in both the promoter-shuffled and corresponding wild-type strains. In order to determine the relationship between protein abundance and cyanamide concentrations, yFYV12 (P_*DDI2*_-*RAD18*) cells were induced with 0–9 mM cyanamide (Fig. 3a). It can be seen that the abundance of the Rad18-5 × FLAG increased linearly after the 0–9 mM cyanamide induction and almost no signal is detected in the absence of cyanamide. The subsequent quantitative analyses show that protein abundance is linearly related to the cyanamide concentration (Fig. 3a). Hence, the protein abundance can be quantitatively regulated by the P_*DDI2*_.

Because *rad18Δ* strain is radiation sensitive, we measured the survival rate of cells after irradiation with different UV doses. The yFYV12 (0/8 mM), yFYV15 (*rad18Δ*) and wild-type cells were respectively spread on YPD plates containing cyanamide, then exposed with different doses of UV. The yFYV12 (P_*DDI2*_-*RAD18*) cells that were treated with 0 and 8 mM cyanamide show similar survival rates as yFYV15 (*rad18Δ*) and wild-type strains, respectively (Fig. 3b).

For the yFYV13 (P_*DDI2*_-*TUP1*) cells, we first observed phenotypic changes at different cyanamide concentrations. The *tup1Δ* strain (yFYV16) was used as a negative control. As shown in Fig. 3c, the yFYV13 cells without cyanamide settle on the bottom of the tube, similar to the yFYV16 strain. Meanwhile, yFYV13 cells induced with 5 mM cyanamide were suspended in the same medium as the wild-type cells. Next, we examined the Tup1-5 × FLAG protein abundance by Western blot after treatment with 0, 2, and 4 mM cyanamide, respectively. We detected an obvious increase in Tup1-5 × FLAG protein level after cyanamide treatment (2 or 4 mM), and no Tup1-5 × FLAG protein expression was detected without inducer cyanamide (Fig. 3d). The results indicate that, by using different concentrations of cyanamide, P_*DDI2*_ is capable of boosting the expression of the target gene to a level that compares to the *tup1Δ* and wild-type strains. Together, these data suggest that the P_*DDI2*_ gene switch can fine-tune the expression of the selected target genes.

### Using *DDI2* promoter controls the expression of essential gene *CDC*6

**Figure 4:**
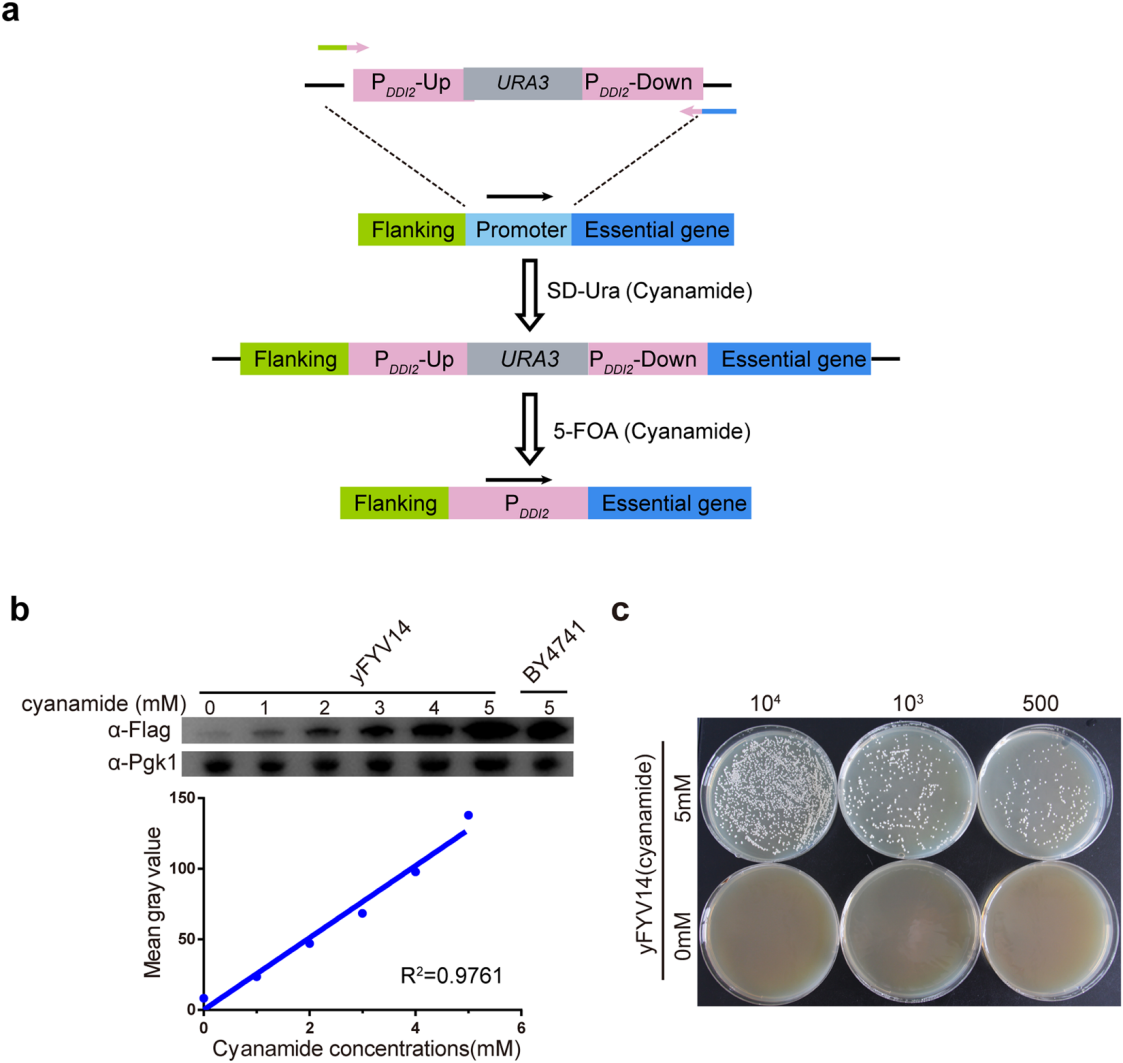
The *DDI2* promoter controls the expression of the essential gene *CDC6* as a safeguard switch. (**a**) Schematic illustration of yeast strain construction replacing the essential gene promoter with P_*DDI2*_. The P_*DDI2*_*-URA3-*P_*DDI2*_ cassette was integrated to replace the native promoter of the essential gene and selected by growing on SD-Ura plates containing cyanamide. Then homologous recombination between these two tandem repeats (P_*DDI2*_) results in the pop-out of *URA3* along with one copy of the P_*DDI2*_. The addition of the cyanamide can ensure cell survival during the replacement of the essential gene promoter. (**b**) yFYV14 (P_*DDI2*_-*CDC6*) cells were induced by 0–5 mM cyanamide for 4 h. Total protein extracts of yFYV14 and BY4741 were measured by western blot analysis using an anti-Flag antibody. The image of blots is cut from the image of Full-length blots, which are presented in Supplementary Fig. S4. The linear equation (y = 25.49x + 0.13) fitted to the change of gray values. **(c**) The yFYV14 strain was induced with 0 or 5 mM cyanamide. Then, a total of 10^4^, 10^3^, and 500 cells of yFYV14 were plated on YPD or cyanamide-containing YPD (5 mM) plates and incubated at 30 °C for 3 days.

To investigate whether the P_*DDI2*_ can linearly and tightly regulate the expression of essential genes as well. Here, we chose *CDC*6 encoding the protein required in the pre-replicative complex formation in DNA replication^44^ as a gene of interest. Compared to other essential gene manipulating method which must transfer the strain with a wild copy of the essential gene to ensure the survival during the modification, we replaced the promoter of *CDC*6 with the P_*DDI2*_ in a simple and efficient method. We designed a P_*DDI2*_-down fragment (Fig. 4a) which partially drove the expression of the essential gene under the induction of cyanamide. Thus, only adding cyanamide can ensure the cell viability during the promoter replacing process, and an extra wild-type copy of the target gene is unnecessary. Based on the recyclable cassette method^37^, we replaced the *CDC*6 native promoter with P_*DDI2*_ (Fig. 4a).

To confirm that the P_*DDI2*_ can tightly control the essential gene (*CDC6*) expression as a gene switch. Overnight cultured yFYV14 (P_*DDI2*_-*CDC6*) cells (with 5 mM cyanamide) were respectively transferred into 5 mM cyanamide or cyanamide-free medium for 4 h. Finally, 10000, 1000, and 500 cells were spread on YPD plates containing 0 and 5 mM cyanamide. As shown in Fig. 4c, no cells survive on YPD plates without cyanamide, which clearly indicates that the P_*DDI2*_ is a tight promoter with no detectable expression leakage. The expression of the Cdc6-5 × FLAG protein was also examined by western blot analysis using an anti-Flag antibody, and a corresponding experimental result is shown in Fig. 4b. It clearly indicates that the protein production corresponds well with the cyanamide concentration (0-5 mM). A small amount of Cdc6-5 × FLAG protein was detected in 0 mM cyanamide sample probably due to intracellular residual cyanamide from the overnight culture. The western blot results show that a linear equation fitted the changes in protein abundance. Together, these results implied that the P_*DDI2*_ can act as a gene switch to control the expression of essential genes.

## Discussion

In this study, we demonstrate that P_*DDI2*_ is a strong inducible promoter. We establish a simple promoter switching method to control the expression of target genes on its genomic locus in budding yeast and quantitatively analyzed the expression of target genes under the control of P_*DDI2*_. Our study suggests the potential of P_*DDI2*_ for linear control of gene expression and as a tight on-off switch.

Four plasmids, containing constitutive promoter *ADH1* or inducible promoters *GAL1*, *CUP1* and *DDI2* linked to the *sfGFP* reporter gene were constructed to compare promoter strengths. The experimental results show that the strength of P_*DDI2*_ is stronger than all P_*ADH1*_, P_*CUP1*_, and P_*GAL1*_ (Fig. 2). It has been reported that inducible promoter P_*CUP1*_ is leaky^45,46^. We also observed a rather high basal expressing level of *sfGFP* even in absence of CuSO_4_ induction (data not shown). Both *GAL1* and *DDI2* are stringent promoters. In our experiments, the *sfGFP* expression under control of these two promoters was undetectable in the absence of corresponding inducers, which is important in cases where background expression is unacceptable, such as the expression of proteins is toxic to yeast cell. Unlike P_*GAL1*_, P_*DDI2*_ did not require replacement of the culture medium during the process of induction, thus total culture time was shorter. Moreover, the induced strength of P_*DDI2*_ was higher than P_*GAL1*_ under the corresponding conditions, and the cost of the cyanamide inducer was less than that of P_*GAL1*_ (galactose)^8^. Accordingly, using P_*DDI2*_ to precisely control gene expression can save a lot of effort and be economical for researchers. More importantly, due to the low price of cyanamide, it has good application prospects in the industrial production that requires high-level induction of target gene with low cost.

Furthermore, the *DDI2* promoter can act as a linear gene switch to regulate the gene expression. As a proof-of-concept, the parental promoters of *RAD18*, *TUP1*, *CDC6* genes were successfully replaced with *DDI2* promoter in the genomic locus, so that the expression of the above three genes are under the control of P_*DDI2*_ as desired. Especially, for regulating an essential gene on its genomic locus, there are several advantages for the P_*DDI2*_-shuffling method over other gene regulated expression systems that function in yeast. Because of the special character of *DDI2* promoter, an extra wild-type copy of the target gene is not required to transfer into the host cell during the promoter switching process, which is simply not possible with other systems. Thus, the counter-selection step for losing the extra copy of the target gene can be omitted to save time and the cost. The linear correlation between cyanamide concentration and expression level is a great benefit for regulating an essential gene, the target essential gene would be expected to produce the designed amount of product to carry out its function.

Recently, the booming development of synthetic biology has made genetically engineered budding yeast strains more widely used in large-scale cultivation and open environments. To prevent unconscious diffusion of genetically engineered strains into natural ecosystems or protect the intellectual property, an important strategy is utilizing on-off switch that strictly controls the expression of essential genes^47^. According to our data, we anticipate that P_*DDI2*_ can be used as such a switch. In our study, the parental promoter of the essential gene *CDC6* was successfully replaced by P_*DDI2*_ in the genomic locus of *S. cerevisiae*. P_*DDI2*_ tight regulated *CDC6* expression and the yeast cells did not survive without the induction of cyanamide. In the future, we anticipate P_*DDI2*_ system could be used as a safeguard switch in the recombinant yeast to achieve the integral biosafety.

We envision that *DDI2* promoter is used for fine-tuning the expression of the interested gene in both the experimental studies and industrial applications. The main concern for the widespread use of *DDI2* promoter could be the low toxicity of inducer cyanamide. The cyanamide can highly induce the expression of *DDI2/3* as MMS which is the DNA-damaging agent. It has been reported that cyanamide has modest toxicity to human^34^, however, no direct evidence has been proved that the toxicity is related to DNA damage. Little is known about the toxicity of cyanamide to yeast, although it has been reported that budding yeast cells lacking the cyanamide hydratase activity displayed an enhanced sensitivity to cyanamide^24^. With the development of chemical synthesis technology, it is expected to design the analogs of cyanamide. Hopefully, the analogous compounds can achieve high-level induction as cyanamide, but with non-toxic to cells.

## Methods

### Plasmid construction

To compare the strength of four promoters, PCR amplified fragment *sfGFP-HIS*_*6*_*-T*_*CYC1*_ was inserted into vector YCplac111 between *EcoR*I and *Sph*I to form YCpLac111-*sfGFP-HIS*_*6*_*-T*_*CYC1*_. Then, copies of P_*ADH1*_, P_*GAL1*_, P_*CUP1*_, and P_*DDI2*_ were cloned into the *BamH*I and *Sph*I sites of the plasmid YCplac111-*sfGFP-HIS*_*6*_*-T*_*CYC1*_ to form four plasmids: YCplac111-P_*ADH1*_-*sfGFP-HIS*_*6*_-T_*CYC1*_, YCplac111-P_*GAL1*_-*sfGFP-HIS*_*6*_*-*T_*CYC1*_, YCplac111-P_*CUP1*_-*sfGFP-HIS*_*6*_-T_*CYC1*_ and YCplac111-P_*DDI2*_-*sfGFP-HIS*_*6*_*-*T_*CYC1*_^37^.

To obtain the P_*DDI2*_-*URA3*-P_*DDI2*_ fragments, we inserted the upstream copy of P_*DDI2*_ into the *BamH*I and *EcoR*I sites of pBluescript-*URA3*^37,48^, then the corresponding downstream P_*DDI2*_ fragment was cloned into the *Hind*III and *Sal*I sites to generate the DUD plasmid (see Supplementary Fig. S1e). To facilitate PCR amplification of the entire DUD cassette, the 3′ end of the upstream promoter and the 5′ end of the downstream copy were truncated to serve as optimized templates.

### Strain construction

The yeast strains used in this study are listed in Table 1. Four plasmids with promoter-*sfGFP* fragments were transformed into yeast strain W303 using a highly efficient LiAc transformation method^49^. The strains were selected on SD-Leu medium (synthetic dextrose medium without leucine) and named yFYV9 (P_*ADH1*_*-sfGFP*), yFYV10 (P_*GAL1*_*-sfGFP*), yFYV11 (P_*DDI2*_*-sfGFP*) and yFYV17 (P_*CUP1*_*-sfGFP*) (Table 1).

**Table 1:** Yeast strains used in this study.

Strains	Genotype
W303	*MATa leu2-3*,*112 trp1-1 can1-1*0*0 ura3-1 ade2-1 his3-11*,*15*
BY4741	*MATa his3Δ1 leu2*Δ*0 met15Δ0 ura3*Δ*0*
yFYV9	W303 with YCplac111-P_*ADH1*_-*sfGFP-HIS*_6_-T_*CYC1*_
yFYV10	W303 with YCplac111-P_*GAL1*_-*sfGFP-HIS*_6_*-*T_*CYC1*_
yFYV11	W303 with YCplac111-P_*DDI2*_-*sfGFP-HIS*_6_-T_*CYC1*_
yFYV12	BY4741 P_*DDI2*_-*RAD18-*5 × *FLAG*::*natMX*6
yFYV13	BY4741 P_*DDI2*_*-TUP1-*5 × *FLAG*::*natMX*6
yFYV14	BY4741 P_*DDI2*_*-CDC6-*5 × *FLAG*::*natMX6*
yFYV15	BY4741 *rad18*:: *natMX6*
yFYV16	BY4741 *tup1*:: *natMX6*
yFYV17	W303 with YCplac111-P_*CUP1*_-*sfGFP-HIS*_*6*_*-*T_*CYC1*_

The reported recyclable cassettes method^37^ was used to replace the native promoters. The promoters of *TUP1*, *RAD18*, and *CDC6* yeast genes were scarlessly replaced by P_*DDI2*_, and the resultant strains were named yFYV12, yFYV13, and yFYV14, respectively (Table 1). DUD recyclable cassettes were PCR amplified using target gene promoter-specific primers linked to the cassette-specific primer sequences^50^ and DUD plasmid as template. The primers used in this study are listed in Supplementary Table S1. A two-step method was used to replace the genomic promoter, and the transformed strain was selected on SD-Ura medium. Subsequently, the *URA3* marker pop-out strain was achieved by using 5-FOA based method^37^. The resulting strains were confirmed by genomic PCR followed by Sanger sequencing, and the *5* × *FLAG* tag was added to the C-terminus of the target genes (*TUP1*, *RAD18*, and *CDC6)* for western blot detection. The *RAD18* and *TUP1* genes were knocked out using the *NAT1* gene, respectively, and the resultant strain was named yFYV15 and yFYV16.

### Fluorescence microscopy

yFYV9 (P_*ADH1*_*-sfGFP*), yFYV10 (P_*GAL1*_*-sfGFP*), yFYV11 (P_*DDI2*_*-sfGFP*) and yFYV17 (P_*CUP1*_*-sfGFP*) cells were cultured in liquid SD-Leu medium overnight at 30 °C. Next morning, 500 µl of the cultures were transferred to tubes containing 5 ml fresh SD-Leu medium (OD600 of 0.4) and incubated for 3 h to an OD600 of 1.0 to 1.2. Next, the yFYV9 (P_*ADH1*_*-sfGFP*) cells were cultured in SD-Leu medium for 3 h, and the yFYV10 (P_*GAL1*_*-sfGFP*) cells were starved in raffinose medium for 3 h, then induced in galactose medium for 3 h. The yFYV11 (P_*DDI2*_*-sfGFP*) cells were cultured in SD-Leu medium with 5 mM cyanamide (C87908-100G, Aldrich) for 3 h and the yFYV17 (P_*CUP1*_*-sfGFP*)cells were cultured in SD-Leu medium with 0.5 mM CuSO_4_ (10008218, ACR) for 3 h. At the end of incubation, 1 OD yeast cells were collected by centrifugation at 2400 g for 2 min, washed once with 1 ml PBS, and resuspended in PBS to final OD600 = 40. Fluorescence microscopy of *sfGFP* expression was performed using an Olympus FV1200 optical microscope (IX81) with a 60× objective lens.

### Flow cytometry

Cells were incubated and treated as described above. The yFYV9 (P_*ADH1*_*-sfGFP*) cells were cultured for different time points (2, 3, and 4 h), the yFYV10 (P_*GAL1*_*-sfGFP*) cells were induced with galactose for different induction time points (2, 3, and 4 h), the yFYV11 (P_*DDI2*_*-sfGFP*) cells were induced with different concentrations of cyanamide (0–8 mM) for 3–5 h, and the yFYV17 (P_*CUP1*_*-sfGFP*) cells were induced with 0.5 mM Cu^2+^ for different induction time points (2, 3, and 4 h). After cell culture, 1 OD cells were collected and resuspended in 1 ml PBS. The fluorescence expression of *sfGFP* in each of the strains was measured using a MoFlo XDP flow cytometry (Beckman). For each strain, approximate 50,000 events were collected to measure fluorescence intensity. Summit 5.2 software was used to analyze the data, and mean fluorescence values were calculated in biological triplicates.

### Irradiation, clumping assay, and cyanamide induces the expression of the essential gene

Cultures of yFYV12, yFYV15, and BY4741 were grown overnight in YPD liquid medium, then transferred to fresh medium for another 4 h. Next, 0/8 mM cyanamide concentrations were added to the yFYV12 cultures. After 4 h, the yeast cells were harvested and diluted to 10^4^ cells/ml. Then, approximately 50 µl of the least diluted cultures were spread on YPD or cyanamide-containing (8 mM) YPD plates in triplicate. The plates were treated with different doses of UV irradiation (0, 10, 20, and 30 J/m^2^) using a UV cross-linking instrument (Analytik Jena’s Model CL-1000), then incubated at 30 °C for at least 4 days.

The yFYV16, BY4741, and yFYV13 cells supplemented with 0 or 5 mM cyanamide were incubated overnight before imaging.

The yFYV14 cells were cultured in liquid YPD with 5 mM cyanamide at 30 °C for 12–16 h. Then, the cells were washed and resuspended in YPD or 5 mM cyanamide-containing YPD medium for 4–6 h. After the cultivation, the yFYV14 cells were harvested and diluted to 10^5^ cells/ml. Approximately 10^4^, 10^3^, or 500 cells were spread on YPD plates (with 0 or 5 mM cyanamide) for 3 days.

Total protein was extracted from yFYV12, yFYV13, and yFYV14 cells induced with different cyanamide concentrations for 4 h, separated by SDS-PAGE, then gels were transferred to a polyvinylidene fluoride membrane and analyzed using immunoblotting. The antibodies used in this study were FLAG tag antibody (M20008M, Abmart) and Pgk1 antibody (ab199438, Abcam). Protein quantification was compared under mean gray value and processed by Image J software (1.50i).

### Statistical analysis

Data were presented graphically using GraphPad Prism software. Mean values and standard deviations were obtained for data analyzed in the study. The T test was applied to assess whether the among groups was significantly different. The Significance of statistical analysis was established at α = 0.01.

## Supplementary information

Fine-tuning the expression of target genes using a DDI2 promoter gene switch in budding yeast

## Data Availability

All data generated or analyzed in this study are included in this published article and its Supplementary Information files.
