# Supplementary material for: Fine-tuning the expression of target genes using a DDI2 promoter gene switch in budding yeast
Source: Sci Rep. 2019 Aug 29;9:12538. doi: 10.1038/s41598-019-49000-8 (PMC6715627; doi:10.1038/s41598-019-49000-8)
Supplement: Supplementary file 1 — Fine-tuning the expression of target genes using a DDI2 promoter gene switch in budding yeast [file 41598_2019_49000_MOESM1_ESM.pdf]

Fine-tuning the expression of target genes using a *DDI2* promoter gene switch in budding yeast

Yong Wang[1,2‡], Kaining Zhang [1,2‡], Hanfei Li[1,2], Xin Xu[1], Huijun Xue[1], Pingping Wang[3], Yu V. Fu[1,2,*]

[1]State Key Laboratory of Microbial Resources, Institute of Microbiology, Chinese Academy of Sciences, Beijing 100101, China

[2]Savaid Medical School, University of Chinese Academy of Sciences, Beijing 100101, China

[3]Qingdao Baihuizhiye Biotech Co.Ltd, Qingdao 266109, China.

‡ These authors contributed equally to this work.

*Address correspondence to Yu V. Fu, fuyu@im.ac.cn.

Supplementary materials

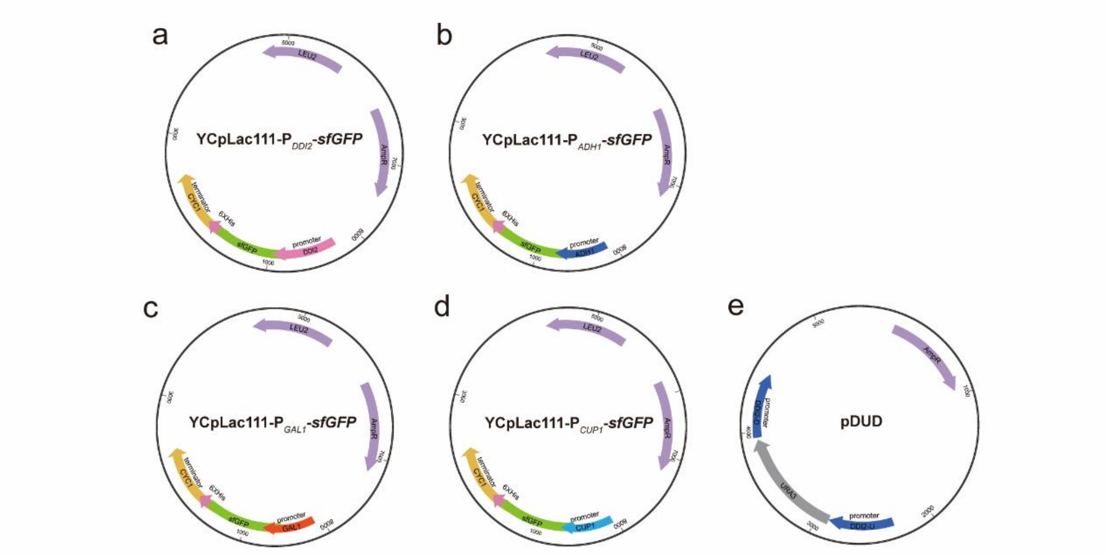

Supplementary Figure S1. Plasmids constructed to compare promoters and for shuffling. (a) Plasmid YCplac111-P$_{DDI2}$-*sfGFP-His*$_6$-T$_{CYC1}$ containing the *DDI2* promoter and reporter gene *sfGFP*. (b) Plasmid YCplac111-P$_{ADH1}$-*sfGFP-His*$_6$-T$_{CYC1}$ containing the *ADH1* promoter and reporter gene *sfGFP.* (c) Plasmid YCplac111-P$_{GAL1}$-*sfGFP-His*$_6$-T$_{CYC1}$ containing the *GAL1* promoter and reporter gene *sfGFP*. (d) Plasmid YCplac111-P$_{CUP1}$-*sfGFP-His*$_6$-T$_{CYC1}$ containing the *CUP1* promoter and reporter gene *sfGFP*. (e) Plasmid DUD containing the entire fragment of the P$_{DDI2}$-*URA3*-P$_{DDI2}$ cassette.

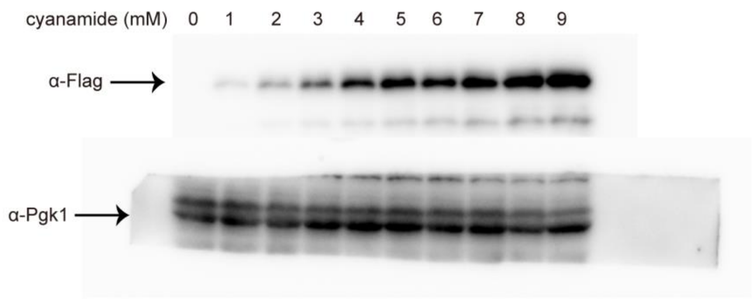

Supplementary Figure S2. Full-length blots of Figure 3(a).

yFYV12 (P$_{DDI2}$-*RAD18*-5×*FLAG*) cells were induced with 0–9 mM cyanamide for 4h,

then the total protein was extracted. The abundance of the Rad18 protein was measured by western blot analysis using an anti-Flag antibody. The Pgk1 protein was used as an internal loading control. The mean gray value of Rad18 in the western blot was quantified using Image J software (1.50i).

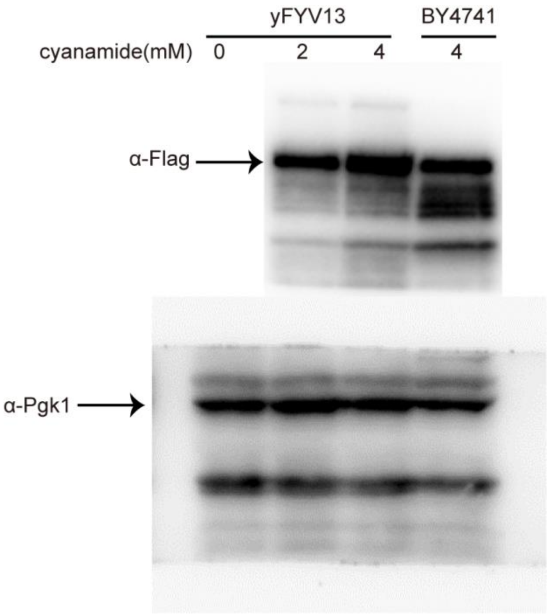

Supplementary Figure S3. Full-length blots of Figure 3(d).

yFYV13 (P_{DDI2}-*TUP1*) cells were induced by cyanamide (0, 2, or 4 mM) for 4 h, then the total protein was extracted from yFYV13 and BY4741. Tup1 protein abundance was measured by western blot analysis using an anti-Flag antibody.

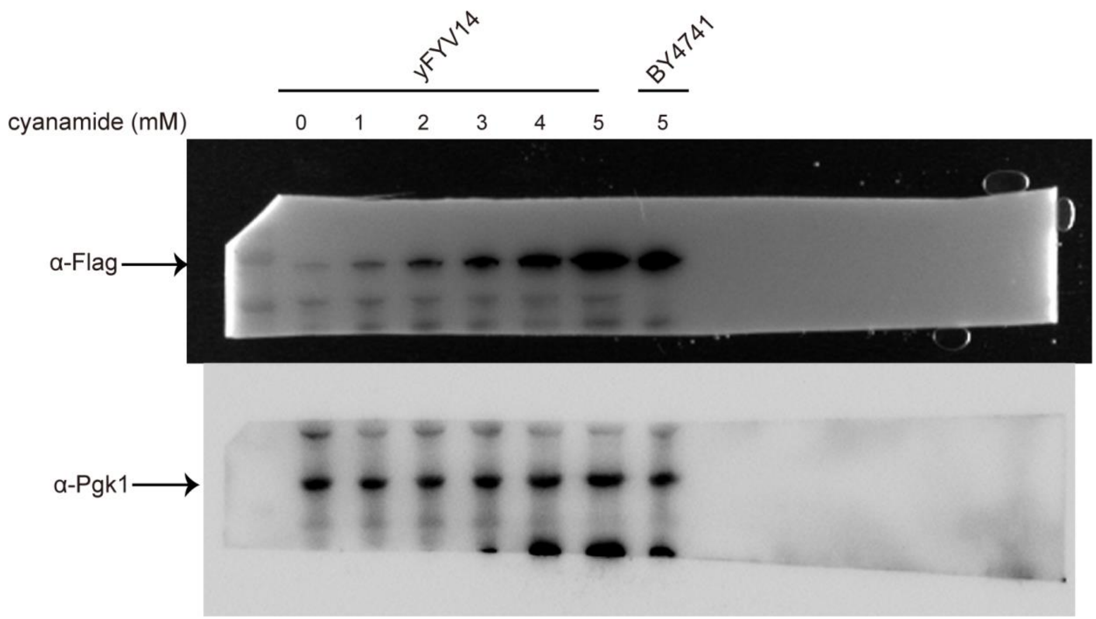

Supplementary Figure S4. Full-length blots of Figure 4(b).

yFYV14 (P_{DDI2}-*CDC6*) cells were induced by 0–5 mM cyanamide for 4h. Total protein extracts of yFYV14 and BY4741 were measured by western blot analysis using an anti-Flag antibody.

Supplementary Table S1. Homologous recombination primers used in this study

Primer	Sequence
P_{DDI2}-*RAD18*-F	5´-AATCACCCTTCCAACGATAGCGGTAATGAAAA GAATTGATCTAAGATAAAACACAGATCGAC-3´
P_{DDI2}-*RAD18*-R	5´-AGTCGTGAAGTCGCTTGCAGTGGTTATTTGGT GGTCCATGATTGATTCTTTTGAAGAGAAGC-3´
P_{DDI2}-*TUP1*-F	5´-TTTCAATGTGACCTTTTTCCAATCATTTTTCAA TGTAAATCTAAGATAAAACACAGATCGAC -3´
P_{DDI2}-*TUP1*-R	5´-ATTCAGCTTATTCTGCGTATTCGAAACGCTGGC AGTCATGATTGATTCTTTTGAAGAGAAGC -3´
P_{DDI2}-*CDC6*-F	5´-GAAATAAATCTCAACTTGAGAGGTTTACATGA CTTTACCTCTAAGATAAAACACAGATCGAC-3´
P_{DDI2}-*CDC6*-R	5´-TCTGATACGCTTAGTTGGAGTTATTGGTATAGC TGACATGATTGATTCTTTTGAAGAGAAGC-3´
